# Are Coronary Calcium-Modifying Techniques Levelling the Playfield?

**DOI:** 10.3390/medicina62040782

**Published:** 2026-04-17

**Authors:** Georgiana Pintea Bentea, Pierre-Emmanuel Massart

**Affiliations:** Department of Cardiology, CHU Namur—Sainte Elisabeth, 5000 Namur, Belgium

**Keywords:** calcified coronary lesions, percutaneous coronary intervention, calcium-modifying technologies

## Abstract

Patients with heavily calcified coronary arteries represent a challenge in percutaneous coronary intervention (PCI), as severe calcification impairs device delivery and limits optimal stent expansion, leading to higher risks of stent thrombosis, restenosis, and adverse clinical outcomes. Approximately 20% of patients undergoing PCI exhibit severe coronary calcification, which independently predicts incomplete revascularization, increased mortality, and higher rates of major adverse cardiovascular events over mid-term follow-up. Recent advances have focused on improving the assessment and management of calcified lesions. Intracoronary imaging modalities, including intravascular ultrasound and optical coherence tomography, allow precise detection and characterization of calcium burden, overcoming the limitations of angiography. These tools play a pivotal role in guiding procedural strategy, enabling tailored selection of calcium-modifying techniques based on lesion morphology, and optimizing stent deployment. Technological innovations have significantly expanded therapeutic options. While non-compliant balloon angioplasty alone is often insufficient, adjunctive devices such as cutting and scoring balloons improve plaque modification in focal disease. Atherectomy techniques, including rotational and orbital systems, are effective for more complex lesions but require technical expertise and carry procedural risks. Intravascular lithotripsy has emerged as a promising, less aggressive modality capable of fracturing deep calcium, while excimer laser atherectomy offers an alternative for resistant lesions. Despite these advances, current evidence supporting calcium-modifying strategies is largely based on procedural outcomes rather than definitive improvements in long-term clinical endpoints. Meta-analyses and randomized trials have not demonstrated clear superiority of any single technique, and most studies remain underpowered. Intriguingly, recent data suggest that outcomes in treated calcified lesions may approximate those of non-calcified disease, raising the hypothesis that these technologies could mitigate the adverse impact of calcification. However, this remains unproven, highlighting the urgent need for adequately powered randomized trials to determine their true clinical benefit.

## 1. Clinical Impact of Coronary Artery Calcification

Patients with heavily calcified coronary arteries face a much tougher journey through percutaneous coronary interventions. For many years, calcified coronary artery disease has represented a major challenge for percutaneous coronary intervention and continues to do so today. Severe coronary calcification limits device deliverability and predisposes to suboptimal stent expansion, which in turn increases the risk of stent thrombosis, in-stent restenosis, target vessel failure, and thus worse clinical outcomes [1]. Pooled data from recent drug-eluting stent studies indicate that about one in five patients have severe coronary calcification, which independently predicts poorer outcomes over a three-year follow-up period [2]. Patients with severely calcified coronary lesions were less likely to achieve complete revascularization compared with those without severe calcification (48% vs. 55.6%, *p* < 0.001). They also had higher mortality rates (10.8% vs. 4.4%, *p* < 0.001). In addition, adverse events were more frequent in the severe calcification group. The combined endpoint of death or myocardial infarction occurred in 22.9% versus 10.9% (*p* < 0.001), and the composite of death, myocardial infarction, or any repeat revascularization occurred in 31.8% versus 22.4% (*p* < 0.001) [2].

In recent years, growing interest in the management of calcified coronary lesions has led to the emergence and refinement of dedicated calcium-modifying technologies aimed at improving lesion preparation and optimizing procedural success [1].

## 2. Role of Intracoronary Imaging

The first step in this endeavour was the development and widespread adoption of intracoronary imaging, which enables earlier and more accurate identification and characterization of calcified coronary stenoses, often underestimated by conventional angiography. Intravascular ultrasound (IVUS) is highly effective for detecting coronary calcium and allows reliable assessment of its distribution, while optical coherence tomography (OCT), with its superior spatial resolution, enables precise quantification of calcium arc and thickness and provides detailed morphological information, including the identification of calcium fractures following lesion preparation [3]. These modalities demonstrate high diagnostic accuracy, with sensitivity and specificity exceeding 90% for dense calcium compared with pathological analysis, whereas fluoroscopy detects only about half of such lesions [3].

Beyond diagnosis, intracoronary imaging plays a central role in decision-making and patient selection. By accurately defining calcium burden, depth, and circumferential extent, imaging helps determine the need for lesion preparation and guides the choice of the most appropriate calcium-modifying strategy. For example, superficial versus deep calcium, focal versus diffuse disease, and lesion length may influence whether balloon-based techniques, atherectomy, or intravascular lithotripsy are preferred. In this way, imaging enables a more tailored, lesion-specific approach rather than a one-size-fits-all strategy.

Furthermore, intravascular imaging is essential throughout the procedure, for not only guiding technique selection but also assessing the adequacy of calcium modification and optimizing stent deployment. It allows confirmation of procedural success through the evaluation of stent expansion, apposition, and the presence of residual calcium-related constraints. As such, imaging-guided strategies contribute to improved procedural precision and may enhance clinical outcomes by supporting appropriate patient and lesion selection.

## 3. Calcium-Modification Techniques

Secondly, substantial advancements have been made in the technologies used to manage calcified coronary stenoses. Initial treatments relied solely on non-compliant balloon angioplasty, which was often inadequate for rigid, heavily calcified lesions. To address this limitation, cutting and scoring balloons—equipped with small blades or scoring elements—were developed to create controlled incisions in the plaque during inflation, thereby improving procedural outcomes in cases of focal calcification [4].

Atherectomy techniques were subsequently introduced to address more complex disease. Rotational atherectomy uses a high-speed, diamond-coated burr to ablate superficial calcium, enabling treatment of tight, heavily calcified lesions that are resistant to balloon dilation. Despite its efficacy, rotational atherectomy is technically demanding, carries a risk of vessel injury, and experienced relatively slow adoption in its early use [5]. Orbital atherectomy operates on a similar principle, but the device rotates in an orbital pattern, gradually sanding the calcium. This allows the treatment of longer lesions with less force while maintaining partial blood flow during the procedure. Nevertheless, it requires precise operator technique and may not be suitable for all lesion morphologies [5].

More recently, intravascular lithotripsy has emerged as a less aggressive alternative, using sonic pressure waves delivered via a balloon to fracture deep and circumferential calcium, thereby improving vessel compliance [5]. It has been rapidly adopted as a preferred option for severe calcification that is resistant to conventional atherectomy or balloon-based techniques [5].

In parallel, excimer laser coronary atherectomy provides an additional option, using pulsed ultraviolet energy to modify plaque and enhance device deliverability, particularly in balloon-uncrossable or undilatable lesions [6].

Together, these evolving technologies have expanded the therapeutic options available for managing calcified coronary disease, allowing a more tailored approach based on lesion characteristics.

## 4. Clinical Outcomes and Evidence Gaps

Although current guidelines recommend the use of calcium-modifying techniques in patients with calcified coronary lesions, these recommendations are largely extrapolated from studies demonstrating improved procedural metrics rather than definitive reductions in hard clinical endpoints. Direct head-to-head comparisons between different modification strategies remain limited, and many available trials are underpowered to detect differences in mortality or major adverse cardiovascular events. Furthermore, most studies focus on surrogate outcomes such as stent expansion or angiographic success, with relatively short follow-up durations. As a result, it remains uncertain whether calcium modification independently improves long-term clinical prognosis compared with conventional lesion preparation, or whether its benefits are primarily procedural. Well-designed, adequately powered randomized trials with extended follow-up are still needed to clarify the true clinical impact of these technologies.

A systematic review and meta-analysis by Andreas Torp Kristensen et al. [7] evaluated randomized trials comparing different PCI strategies for moderately or severely calcified coronary lesions. The authors analyzed 31 trials (8453 patients), including 16 lesion-preparation techniques (e.g., atherectomy, lithotripsy, specialty balloons), focusing on all-cause mortality and serious adverse events. Overall, no lesion preparation technique demonstrated superiority in reducing mortality or serious adverse events. However, most comparisons were underpowered and based on low-certainty evidence, indicating persistent clinical equipoise and the need for adequately powered randomized trials to determine the optimal strategy for calcified lesions [7].

Interestingly, the Disrupt CAD III Study reported a 1-year composite endpoint—cardiac death, myocardial infarction (MI), or ischemia-driven target vessel revascularization—of 13.8% using intravascular lithotripsy [8], while the ROLLING STONE Study found a similar 14.3% at 12 months in a real-world population undergoing rotational or orbital atherectomy [5]. Strikingly, these rates are nearly identical to the 13.9% major adverse cardiovascular event rate seen in patients with coronary stenosis without severe calcification in Bourantas et al.’s meta-analysis [2].

This stands in glaring contrast to the long-term data from the largest patient-level meta-analysis on drug eluting stent outcomes, where severe calcification drove a 44% higher risk of cardiac death, a 23% higher risk of target-vessel MI, and a 21% higher rate of target lesion failure compared with non-calcified lesions [9].

## 5. Conclusions and Future Directions

Taken together, these observations hint—almost provocatively—that calcium-modifying strategies may blunt the disadvantage of severe calcification, essentially “levelling the playing field”. Yet this tantalizing suggestion remains entirely indirect and this concept should be considered at most hypothesis-generating. Importantly, comparisons across studies—particularly between calcified and non-calcified populations—are indirect and inherently limited. These analyses are subject to significant heterogeneity in patient selection, lesion characteristics, procedural techniques, and operator experience. Moreover, selection bias may play a substantial role, as patients undergoing calcium-modifying strategies are often carefully selected or treated in specialized centres, potentially influencing outcomes. We still lack robust, randomized trials powered for clinical endpoints to confirm whether these techniques genuinely translate into improved long-term outcomes, leaving a critical gap in evidence that demands urgent attention.
